# Targeting protein methylation in pancreatic cancer cells results in KRAS signaling imbalance and inhibition of autophagy

**DOI:** 10.1038/s41419-023-06288-9

**Published:** 2023-11-23

**Authors:** María F. Montenegro, Román Martí-Díaz, Ana Navarro, Jorge Tolivia, Luis Sánchez-del-Campo, Juan Cabezas-Herrera, José Neptuno Rodríguez-López

**Affiliations:** 1 Department of Biochemistry and Molecular Biology A, School of Biology, University of Murcia, Instituto Murciano de Investigación Biosanitaria (IMIB), Murcia, Spain; 2 Departamento de Morfología y Biología Celular, Universidad de Oviedo, Grupo GECYEN del Instituto de Investigación Sanitaria del Principado de Asturias (ISPA), Oviedo, Asturias Spain; 3 Molecular Therapy and Biomarkers Research Group, University Hospital Virgen de la Arrixaca, IMIB, Murcia, Spain

**Keywords:** Targeted therapies, Drug development

## Abstract

Pancreatic cancer cells with mutant KRAS require strong basal autophagy for viability and growth. Here, we observed that some processes that allow the maintenance of basal autophagy in pancreatic cancer cells are controlled by protein methylation. Thus, by maintaining the methylation status of proteins such as PP2A and MRAS, these cells can sustain their autophagic activity. Protein methylation disruption by a hypomethylating treatment (HMT), which depletes cellular S-adenosylmethionine levels while inducing S-adenosylhomocysteine accumulation, resulted in autophagy inhibition and endoplasmic reticulum stress-induced apoptosis in pancreatic cancer cells. We observed that by reducing the membrane localization of MRAS, hypomethylation conditions produced an imbalance in KRAS signaling, resulting in the partial inactivation of ERK and hyperactivation of the PI3K/AKT–mTORC1 pathway. Interestingly, HMT impeded CRAF activation by disrupting the ternary SHOC2 complex (SHOC2/MRAS/PP1), which functions as a CRAF-S259 holophosphatase. The demethylation events that resulted in PP2A inactivation also favored autophagy inhibition by preventing ULK1 activation while restoring the cytoplasmic retention of the MiT/TFE transcription factors. Since autophagy provides pancreatic cancer cells with metabolic plasticity to cope with various metabolic stress conditions, while at the same time promoting their pathogenesis and resistance to KRAS pathway inhibitors, this hypomethylating treatment could represent a therapeutic opportunity for pancreatic adenocarcinomas.

## Introduction

The mitogen-activated protein kinase (MAPK) signaling pathway plays an essential role in the control of cell proliferation, differentiation, and survival under physiological conditions [1]. Mutations in some of its components, such as KRAS, produce an oncological activation of this pathway that significantly contributes to cell transformation and is unquestionably involved in tumor progression [2]. KRAS is activated by specific mutations in ~30% of human tumors and is one of the preferential mutations in pancreatic ductal adenocarcinoma (PDAC). For these reasons, this pathway has been the subject of intense research over the past decades, with the aim of identifying components that can be used as therapeutic targets in cancer treatment. However, despite the recent development of drugs that block KRAS^G12C^, the majority of KRAS oncoproteins remain undruggable [3]. Therefore, current strategies to block KRAS-mutant tumors have primarily focused on inhibiting downstream MAPK signaling. Several selective inhibitors of MEK and ERK have been developed as potential agents to combat KRAS-mutated cancers [4, 5]. However, other studies have concluded that MAPK pathway inhibition in KRAS-mutated cancers triggers a resistance mechanism through the induction of protective autophagy [6–8]. Indeed, MEK1/2 inhibition leads to activation of the LKB1 → AMPK → ULK1 signaling axis, a key regulator of autophagy. Therefore, combination therapy of KRAS pathway inhibitors and autophagy inhibitors could represent a new strategy to treat KRAS-mutated cancers [6–8].

Methylation is crucial for many cellular activities, and differences in intracellular S-adenosylmethionine (SAM) concentrations and the SAM/S-adenosylhomocysteine (SAH) ratio affect many biological processes, from the epigenetic regulation of gene expression to the control of DNA damage response pathways [9–11]. These evidences, accumulating over the past 10 years, point to the methionine cycle as a potential therapeutic target. Therapies based on the suppression of the methionine cycle would result in the indirect inhibition of both DNA and protein methylation, although the particular inhibition of DNA or protein methylases primarily results in specific unmethylated products [12]. This comprehensive approach may be desirable due to the vast number of genes and pathways that DNA and protein methylation can affect. When the many effects on cellular physiology are added together, the effects of hypomethylation treatments are expected to have a positive overall therapeutic effect. Indeed, this nonspecificity can be viewed as advantageous where multiple defects are corrected simultaneously. For instance, we observed that a combined therapy designed to uncouple adenosine metabolism using dipyridamole (DIPY) in the presence of a new synthetic antifolate, 3-*O*-(3,4,5-trimethoxybenzoyl)-(-)-catechin (TMCG), effectively and simultaneously blocked both the folic cycle and the methionine cycle in breast cancer cells, making these cells more sensitive to radiotherapy [10]. Here, in the case of pancreatic cancer cells, we observed that blocking the epigenetic machinery resulted in autophagy inhibition and endoplasmic reticulum (ER) stress-induced apoptosis. The objectives of this study were to understand the role of protein methylation on the regulation of autophagy in pancreatic cancer and investigate how its modulation could generate new therapies against this type of cancer [13, 14].

## Results

### HMT inhibits basal autophagy in pancreatic cancer cells

**Fig. 1:**
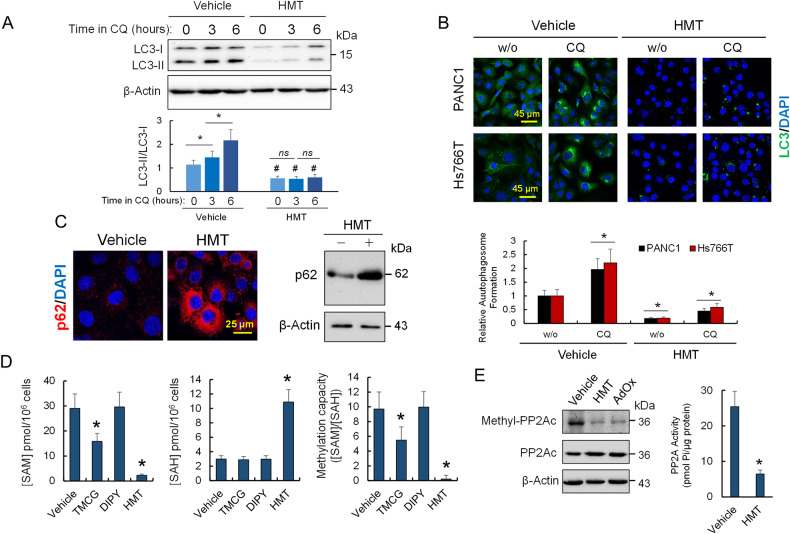
HMT inhibits basal autophagy and decreases the methylation capacity of pancreatic cancer cells. **A** Western blot experiment showing a time-course analysis of LC3-II/LC3-I. PANC1 cells were treated with vehicle or HMT for 48 h followed by treatment with CQ (40 µM) for the indicated times. *P < 0.05. ns = not significant. Differences between vehicle and HMT-treated samples at each time were found significant (^#^*P* < 0.05). **B** Pancreatic cancer cells were treated with vehicle or HMT for 48 h followed (or not) by treatment with CQ (40 µM) for an additional 24 h. Accumulated autophagosomes were evaluated by LC3 staining. *P < 0.05 when compared with vehicle-treated cells without CQ (w/o). **C** Accumulation of p62 was analyzed by confocal microscopy (left) and Western blot (right) in PANC1 cells after 72 h of treatment with vehicle or HMT. **D** Effect of HMT and individual treatments [TMCG (10 µM) or DIPY (5 µM)] on the methylation capacity of PANC1 cells. Metabolite determination was performed 72 h after treatment. **E** Effect of HMT and AdOx (20 µM) on the methylation status (at L309) of the catalytic PP2Ac subunit. The histogram shows the effect of HMT on PP2A activity. Protein and activity analysis was carried out 72 h after each indicated treatment. In this figure, blots are representative of three independent experiments. Error bars show the mean ± SD. *P < 0.05 compared with vehicle-treated controls.

Time-course of autophagy blockage using CQ allows monitoring the autophagy flux [15]. As expected, and in accordance with the elevated levels of basal autophagy, untreated PANC1 cells showed high steady-state levels of LC3-II (Fig. 1A). In control condition, autophagic flux blockade by CQ led to the accumulation of LC3-II due to the buildup of newly formed autophagosomes (Fig. 1A). In contrast, in cells pre-treated with a combination of TMCG and DIPY (HMT), LC3-II levels (compared with LC3-I levels) did not significantly increase in presence of CQ (Fig. 1A), suggesting that HMT pre-treatment had already blocked the autophagic flux. Similar results were obtained by LC3B immunocytochemistry where the treatment of pancreatic cancer cells with HMT efficiently prevented autophagosome formation in cells subjected to CQ treatment (Fig. 1B), indicating that HMT inhibited autophagy at an early state. On the other hand, the accumulation of p62/SQSTM1 in response to HMT also indicated the blockage of autophagy in PANC1 cells (Fig. 1C) [16].

### HMT decreases the cellular methylation capacity of pancreatic cancer cells

Although treating PANC1 cells exclusively with DIPY did not significantly modify the intracellular levels of SAM and SAH, we observed that including TMCG in the treatment produced a consistent and significant decrease in the methylation capacity of PANC1 cells (Fig. 1D). Interestingly, cells treated with the HMT combination showed not only a significant decrease in SAM but also accumulation of higher levels of SAH (Fig. 1D), which is in accordance with the proposed demethylating activity of this drug combination [12]. To determine the impact of decreased methylation capacity in PANC1 cells after HMT treatment, we focused on protein phosphatase 2A (PP2A), a trimeric serine/threonine phosphatase regulated by protein methylation [17]. Although PP2A was found methylated at its catalytic subunit during basal autophagy, we found that HMT significantly induced demethylation of this protein, in the same way that adenosine-2,3-dialdehyde (AdOx), an inhibitor of SAH hydrolase (Fig. 1E). HMT-dependent PP2A demethylation was also accompanied by a substantial time-dependent reduction in PP2A activity (Fig. 1E). These results could be important in the context of pancreatic cancer. A recent investigation revealed that “autophagy-addicted” cells are dependent on high phosphatase activity and that the regulation of autophagy could be coordinated by the action of mTOR complex-1 (mTORC1) and PP2A [18, 19].

### HMT restores cytoplasmic retention of MiT/TFE proteins in PDAC

**Fig. 2:**
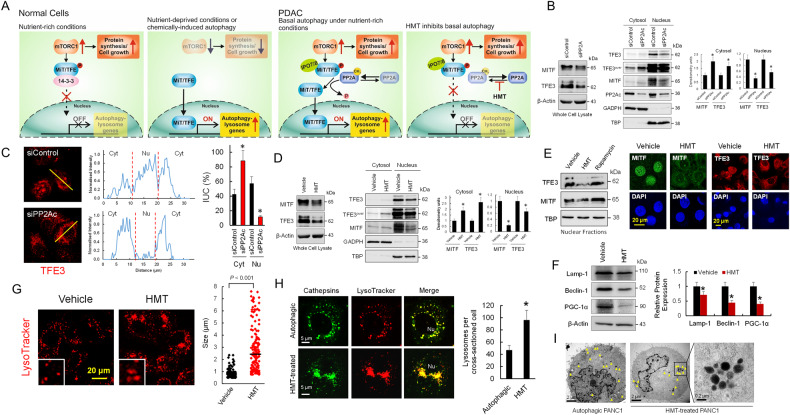
HMT inhibited nuclear translocation of MiT/TFE proteins in PDAC. **A** In normal cells and under nutrient-rich conditions, MiT/TFE proteins are repressed by mTORC1 via phosphorylation and remain in the cytosol; however, upon starvation, mTORC1 is inactivated and MiT/TFE proteins enter the nucleus, where they activate autophagy-lysosome gene transcription. Interestingly, this mechanism was found to be decoupling in PDAC [21]. Although overexpression of the nucleocytoplasmic transporter importin (IPO-7/8) was proposed to facilitate the nuclear translocation of MiT/TFE, the high PP2A activity found in autophagic PDAC cells could also explain the MiT/TFE factors decoupling mechanism. The possible mechanism by which HMT inhibits basal autophagy in PDCA is also depicted. **B** Efficient PP2Ac silencing in PANC1 cells promotes cytosolic accumulation of MITF and TFE3 factors (*P < 0.05 when compared with siControl transfected cells). Blots were overexposed (TFE3^over^) for cytosolic TFE3 quantification. **C** Fluorescence microscopy showing the effect of PP2Ac silencing on the localization of TFE3 in Hs766T cells. The line bar (30 µm) was used as a template for fluorescence intensity determination. Relative occupancy of TFE3 in the nucleus (Nu) and cytosol (Cyt) was calculated by analysis of the fluorescence intensity under the curve (IUC) in each compartment. *P < 0.05 when compared with siControl transfected cells. **D** HMT promotes cytosolic accumulation of MITF and TFE3 factors in PANC1 cells (*P < 0.05 when compared with vehicle-treated cells). Blots were overexposed (TFE3^over^) for cytosolic TFE3 quantification. **E** HMT avoids nuclear translocation of TFE3 and MITF in Hs766T cells. Cells were treated with vehicle or HMT (72 h) or rapamycin (0.2 µM; 120 min). Cell extracts were analyzed by Western blotting for the indicated proteins (left panel). Nuclear localization of MITF and TFE3 was also evaluated by confocal microscopy (right panels) in Hs766T cells treated with vehicle or HMT (72 h). **F** Relative expression of proteins 72 h after each indicated treatment. Histogram represents the relative amount of proteins with respect to actin. *P < 0.05 when compared with vehicle-treated cells. **G** HMT treatment (72 h) causes aberrant lysosomal morphology and increased size as shown by LysoTracker staining. Inset: magnified view. Graph (right) quantification of lysosome diameter in HMT (*N* = 173) and vehicle-treated cells (*N* = 169). Bar, mean. **H** Confocal microscopy showing lysosome number and distribution in autophagic cells and HMT-treated cells. Lysosomes were stained with LysoTracker-Red DND-99 and an iABP Pan cathepsin probe (green). Differences in the number of lysosomes were significant (*P < 0.01) when compared with untreated autophagic cells (graph, right). **I** TEM showing lysosome localization in autophagic cells and HMT-treated cells. The arrow indicates the position of lysosomes. The boxed area contains representative lysosomes at 60,000× magnification. Lysosomes were randomly distributed in the cytoplasm of autophagic PANC1 cells, but HMT-treated cells showed grouped lysosomes.

mTORC1 has been identified as a master regulator of autophagy since inhibition of mTORC1 is required to initiate the autophagy process [20]. When active, this complex phosphorylates and controls the subcellular localization of various transcription factors to suppress the expression of genes necessary for autophagosome formation and lysosome biogenesis (Fig. 2A). In this sense, Perera et al. [21] clearly stated that, as a part of the pathogenesis of PDAC, MiT/TFE transcription factors (MITF, TFE3, and TFEB) are decoupled from regulatory mechanisms that control their cytosolic retention (Fig. 2A). Recent investigations, suggest that high protein phosphatase PP2A activity in PDAC could be a compatible mechanism for MiT/TFE cytosolic/nuclear dysregulation. High PP2A activity has been found not only necessary to maintain autophagy in PDAC [18] but also to dephosphorylate TFE proteins at several serine residues facilitating their activation (Fig. 2A) [22]. Overall, these data suggest a critical mechanism to promote MiT/TFE activation by stimulating PP2A without suppressing mTORC1 activity.

To check whether this mechanism, involving PP2A, is operative in PDAC, we silenced the catalytic subunit of PP2A in Hs766T and PANC1 cells and analyzed the cellular localization of MITF and TFE3 (Fig. 2B, C). As observed in these figures, the lack of PP2A activity in pancreatic cancer cells clearly resulted in a reduction of nuclear MITF and TFE3, while significantly increased cytoplasmic retention of both proteins. Interestingly, and in agreement with the effects of HMT on PP2A methylation and activity (Fig. 1E), this treatment clearly reduced nuclear accumulation of MITF and TFE3 in pancreatic cancer cells (Fig. 2D, E). Consequently, HMT reduced the expression of Lamp-1, Beclin-1 and PGC-1α [23, 24], some of the autophagic/lysosomal genes transactivated by MiT/TFE proteins (Fig. 2F). We also found that treatment with HMT resulted in increased lysosome diameter in PANC1 cells (892.1 ± 34.2 versus 2859 ± 145 nm for vehicle and HMT treatments, respectively) (Fig. 2G), which has been associated with lysosomal stress and defective proteolysis after depletion of MiT/TFE proteins across a series of PDAC cell lines [21]. Paradoxically, we also observed an increased number of lysosomes in HMT-treated cells when compared with untreated cells (Fig. 2H). Lysosomes are considered dynamic organelles and MiT/TFE proteins are key transcriptional regulators of lysosomal homeostasis and autophagy induction; therefore, defective synthesis of autophagic vacuoles under HMT treatment could lead to the accumulation of pre-existing lysosomes in PDAC cells. This hypothesis could be compatible with the observed mistrafficking/accumulation of lysosomes under HMT treatments in PANC1 cells (Fig. 2I).

### HMT activates mTORC1 activity while prevents ULK1 activation

**Fig. 3:**
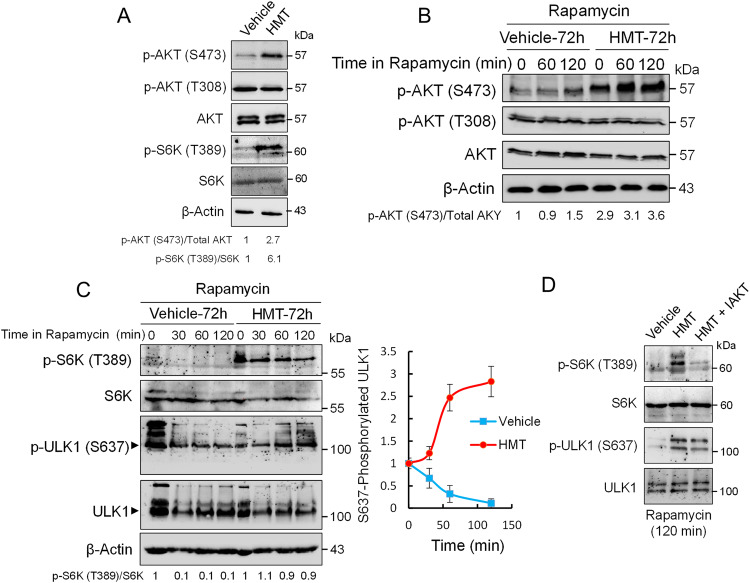
HMT activates AKT/mTORC1 and prevents ULK1 activation in PANC1 cells. **A** Western blot showing the effects of HMT on AKT and S6K phosphorylation under basal autophagy. **B** Western blots showing the effect of HMT on AKT phosphorylation after rapamycin (0.2 µM) treatment. Before rapamycin treatment, the cells were treated with vehicle or HMT for 72 h. **C** Western blots showing the effects of HMT on S6K and ULK1 phosphorylation under rapamycin-induced autophagy. The graph represents the level of phosphorylated ULK1 (at S637) at the indicated times after rapamycin (0.2 µM) exposure. Before rapamycin treatment, the cells were treated with vehicle or HMT for 72 h. Error bars show the mean ± SD. **D** Western blots showing the effects of HMT and HMT/IAKT on S6K and ULK1 phosphorylation under rapamycin-induced autophagy (0.2 µM). Before rapamycin treatment, the cells were treated with vehicle, HMT, or a combination of HMT and IAKT (10 µM) for 72 h. In this figure, the blots shown are representative of three independent experiments.

The phosphorylation of AKT at regulatory residues T308 and S473 leads to its full activation [25]. Here, we observed that HMT treatment markedly increased AKT phosphorylation, mainly at residue S473, in both basal and rapamycin-induced autophagy (Fig. 3A, B). AKT is known to stimulate mTORC1 activity; therefore, these results indicated that HMT-dependent activation of AKT might prevent the inhibition of mTORC1 under autophagic stimuli. To test this hypothesis, we assayed the phosphorylation status of S6K, a well-known substrate of mTORC1. As observed in Fig. 3A, HMT highly promoted the phosphorylation of S6K. Interestingly, treating PANC1 cells with the specific mTORC1 inhibitor rapamycin was not sufficient to inhibit S6K phosphorylation in HMT-treated cells (Fig. 3C). Whether the activation of mTORC1 in HMT-treated cells was specifically due to HMT-dependent activation of AKT was investigated by cotreating PANC1 cells with HMT and a specific inhibitor of AKT (IAKT). The presence of IAKT during HMT treatment was sufficient to completely inhibit S6K phosphorylation in rapamycin-treated PANC1 cells (Fig. 3D).

Dephosphorylation of ULK1 at S637 is required for autophagy induction [26], but steady-state levels of ULK1-S637 might depend on both phosphorylation by mTORC1 and dephosphorylation by PP2A [18, 19]. Since HMT treatment could promote ULK1 phosphorylation at S637 by activating mTORC1 and/or by inactivating PP2A, it was tempting to speculate that HMT treatment could result in the inhibition of ULK1-dependent autophagy. To test this hypothesis, we monitored the UKL1 phosphorylation status at residue S637 (Fig. 3C). We observed that the kinetics of UKL1 dephosphorylation in PANC1 cells were rapid under rapamycin-induced autophagy; however, the presence of HMT induced a time-dependent increase in S637-phosphorylated ULK1. Since rapamycin failed to inhibit mTORC1 in the presence of HMT, to understand the real contribution of the inhibition of PP2A by HMT on steady-state levels of ULK1-S637, we carried out experiments with rapamycin in the presence of HMT and IAKT. We observed that even in the absence of mTORC1 activity (as indicated by the lack of S6K phosphorylation in HMT/IAKT-treated cells), HMT stabilized the S637-phosphorylated state of ULK1 (Fig. 3D). These results further confirm that the mTORC1/UKL1 autophagy pathway is controlled by protein methylation in pancreatic cancer cells. The inhibition of PP2A by HMT not only suppressed autophagy by maintaining functional mTORC1 activity but also inhibited ULK1-dependent autophagy by stabilizing the inactive phosphorylated form of ULK1.

### HMT dysregulates oncogenic MAPK signaling by blocking CRAF activation

**Fig. 4:**
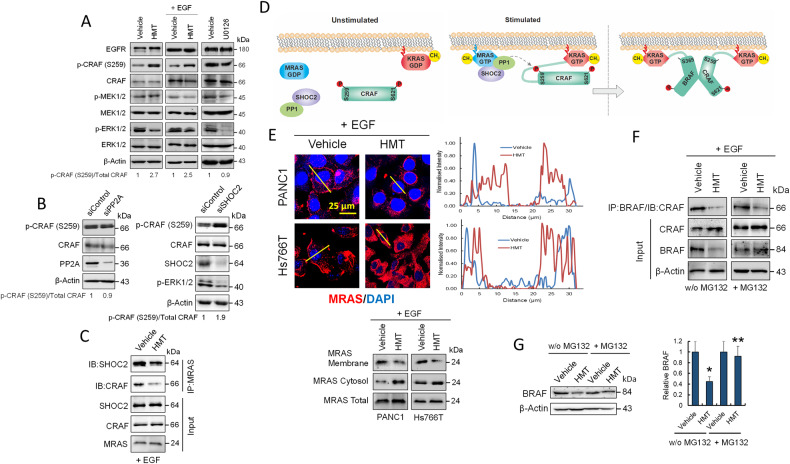
Effects of HMT on MAPK signaling pathway. **A** Western blots showing the effects of HMT with and without EGF stimulation, and U0126 (10 µM; 24 h) on several components of the MAPK pathway in PANC1 cells. **B** Effect of PP2A (left panel) or SHOC2 (right panel) silencing on CRAF phosphorylation at S259 in PANC1 cells. The silencing effect was determined according to the corresponding siControl. **C** HMT reduces coimmunoprecipitation of MRAS with SHOC2 and CRAF. Total PANC1 extracts (1 mg) were immunoprecipitated using anti-MRAS, and bound proteins were immunoblotted with SHOC2 or CRAF antibodies. **D** A model for CRAF activation following dephosphorylation of S259 by the SHOC2 complex. **E** HMT modulates the localization of MRAS in PANC1 and Hs766T cells as determined by confocal microscopy (line bars were used as a template for fluorescence intensity determination) and Western blot analysis. **F** The effect of HMT on CRAF/BRAF heterodimerization in the absence or presence of MG132. For assays without MG132, PANC1 cells were treated with vehicle or HMT for 72 h. For assays in the presence of MG132 (10 µM), cells were treated with vehicle or HMT for 67 h, followed by treatment with MG132 for an additional 5 h. Total cell extracts (1 mg) were immunoprecipitated using anti-BRAF, and bound proteins were immunoblotted with a CRAF antibody. **G** Effect of HMT on BRAF stability in PANC1 cells in the presence or absence of MG132 (10 µM) as indicated in the previous panel. Total cell extracts were used for BRAF quantification. *P < 0.05 compared with vehicle-treated cells; **P < 0.05 compared with HMT-treated cells in the absence of MG132.

Protective autophagy in KRAS-mutant cancers is an attractive target for PDAC [6–8]. Therefore, we investigated whether HMT interfered with the RAF–MEK–ERK MAPK cascade and observed that HMT attenuated, but did not completely abolish, the constitutive phosphorylation of MEK1/2 and ERK1/2 in PANC1 cells (Fig. 4A). Although HMT did not visibly affect EGFR expression in PANC1 cells (Fig. 4A), we observed that this treatment modulated the phosphorylation of CRAF at residue S259 in both EGF-stimulated and nonstimulated cells. In both cases, in the presence of HMT, there was a clear accumulation of inactive CRAF-S259. Since PP2A holoenzymes were found to associate with CRAF, early studies proposed that PP2A activity might be a prerequisite for CRAF activation by removing inhibitory phosphorylation at S259 [27]. However, the fact that this proposed role was mainly based on the use of okadaic acid, a phosphatase inhibitor that also inhibits PP1, together with recent studies that identified the SHOC2–MRAS–PP1 complex as a specific CRAF-S259 phosphatase [28, 29], has reopened the debate on the role of PP2A in CRAF activation [30]. Here, we observed that PP2A activity was expendable during CRAF activation because silencing PP2Ac did not significantly affect the levels of CRAF-S259 phosphorylation in PANC1 cells (Fig. 4B). However, silencing SHOC2 in PANC1 cells did produce an accumulation of CRAF-S259 and an attenuation in the activation of ERK1/2, emulating the effects produced by HMT (Fig. 4B).

### HMT disrupts SHOC2 complex formation by blocking MRAS interaction with the plasma membrane

MRAS GTPase, a close relative of RAS oncoproteins, interacts with SHOC2 and PP1 to form a heterotrimeric holoenzyme (SHOC2 complex) that dephosphorylates the inhibitory S259 site on CRAF kinase [28–30]. Therefore, we examined the interactions of MRAS with SHOC2 and CRAF in PANC1 cell extracts. As shown in Fig. 4C, HMT significantly reduced such interactions, as determined by immunoprecipitation of MRAS and blotting with both SHOC2- and CRAF-specific antibodies. MRAS membrane localization is essential for SHOC2 complex activity toward CRAF in vivo (Fig. 4D) [29]. Interestingly, it has been reported that the accumulation of SAH under hypomethylating conditions inhibits isoprenylcysteine carboxyl methyltransferase (ICMT) [31]. Since the carboxyl methylation of RAS proteins by ICMT is a crucial step for proper plasma membrane localization, the inhibition of ICMT by antifolates results in the mislocalization of RAS proteins to cytosolic compartments [31]. Having observed that treating PANC1 cells with HMT increased the SAH/SAM ratio (Fig. 1D), we analyzed the localization of MRAS in EGF-stimulated pancreatic cancer cells. Confocal microscopy and Western blotting of membrane and cytosolic proteins revealed an enrichment of MRAS in cytosolic compartments after exposure to HMT (Fig. 4E), which could explain the lack of activation of the SHOC2 complex in these treated cells. Although ICMT inhibition might also affect other RAS proteins, such as KRAS, its constitutive activation in KRAS-mutant cancers could make it less sensitive to protein methylation disruption (Fig. 4D).

### HMT prevents heterodimerization of CRAF–BRAF and leads to BRAF destabilization

In the absence of SHOC2, EGF-stimulated BRAF–CRAF heterodimerization is strongly impaired [28]. Therefore, to further explore the effect of HMT on the KRAS pathway, we analyzed the interactions between BRAF and CRAF in PANC1 cells (Fig. 4F). We used equal amounts of vehicle- or HMT-treated cell extracts for immunoprecipitation assays with an anti-BRAF antibody. Although the amount of immunoprecipitated CRAF decreased in HMT-treated extracts, we observed a significant drop in BRAF levels at the corresponding HMT input, indicating that HMT compromised BRAF stability. To confirm this, we analyzed the amount of BRAF in cells treated with vehicle or HMT in the presence or absence of MG132. As observed in Fig. 4G, proteasome inhibition by MG132 restored BRAF levels in HMT-treated cells. Therefore, to analyze whether HMT impaired the BRAF–CRAF interaction, we used cell extracts treated with vehicle or HMT in the presence of MG132 for immunoprecipitation assays with an anti-BRAF antibody. As shown in Fig. 4F, HMT prevented BRAF–CRAF heterodimerization in PANC1 cells.

### HMT suppresses MAPK but activates PI3K activity in KRAS-mutant pancreatic cancer cells

**Fig. 5:**
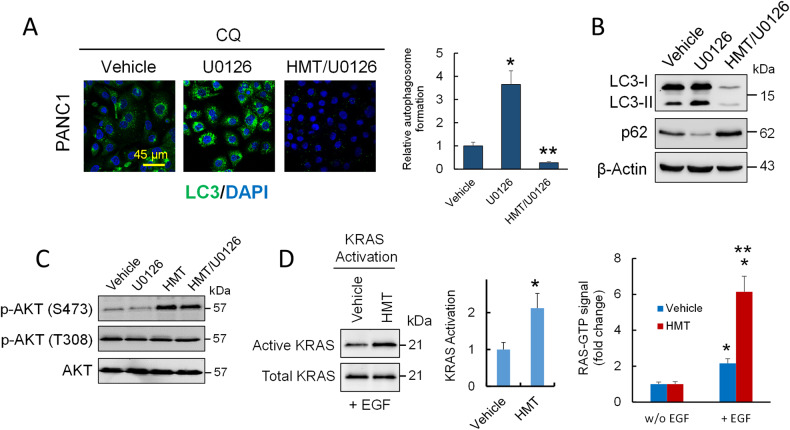
HMT suppresses MAPK but activates PI3K activity. **A** Accumulated autophagosomes in PANC1 cells subjected to the indicated treatments were evaluated by LC3 staining. For these assays, PANC1 cells were treated with vehicle or U0126 (10 µM) for 24 h followed by treatment with CQ (40 µM) for an additional 6 h. For combined HMT/U0126 treatment, HMT-pretreated cells (24 h) were treated with U0126 and CQ as described above. *P < 0.05 compared with vehicle-treated cells. **P < 0.05 compared with U0126-treated cells. **B** Effect of HMT on U0126-induced protective autophagy. Representative Western blot of LC3B and p62 from PANC1 cells treated with vehicle, U0126, or HMT/U0126 as indicated above and then treated with CQ (40 µM) for an additional 6 h. **C** Western blots showing the effects of U0126 and/or HMT treatments on AKT phosphorylation. For individual treatments, PANC1 cells were treated with U0126 (10 µM; 24 h) or HMT (48 h). For combined HMT/U0126 treatment, HMT-pretreated cells (24 h) were treated with U0126 (10 µM) for an additional 24 h. **D** The effect of HMT on RAS activation in PANC1 cells was determined by two independent methods. The cells were treated with vehicle or HMT for 72 h and then stimulated or not with EGF. Left panel: the active form of RAS was carefully isolated from endogenous lysates using a KRAS activation assay kit that makes use of CRAF RBD agarose beads. Using a polyclonal anti-KRAS antibody, Western blot analysis was used to identify the precipitated GTP-KRAS. Error bars show the mean ± SD. *P < 0.05 compared with vehicle-treated cells. Right panel: after EGF stimulation, RAS activation was assayed using an ELISA-based assay for the quantification of GTP-bound RAS and normalized to its level in unstimulated cells. Error bars indicate the SD. *P < 0.05 compared with the respective unstimulated cells. **P < 0.05 compared with vehicle + EGF-treated cells.

Although both HMT and U0126 disrupted the RAF–MEK–ERK pathway, they showed opposing effects on autophagy; while HMT inhibited autophagic flux (Fig. 1A), U0126 induced protective autophagy (Fig. 5A, B). In fact, we observed that HMT inhibited protective autophagy induced by U0126 in PANC1 cells (Fig. 5A, B). Differences in AKT activation after HMT and U0126 treatments could explain the differential response of PANC1 cells to these treatments (Fig. 5C). Although several components of the pathway controlling AKT can be affected by the methylation status of the cell, we observed that after EGF stimulation, there was an increase in KRAS activation in those samples that were pretreated with HMT compared to those pretreated with only the vehicle (Fig. 5D). Because the inhibition of SHOC2 expression suppresses MAPK but not PI3K activity in tumor cells with RAS gene mutations [30], these observations could partly explain the overactivation of the PI3K pathway in cells treated with HMT, which could contribute to increased phosphorylation of AKT after the inhibition of the SHOC2 complex.

### HMT induces ER stress and cell death

**Fig. 6:**
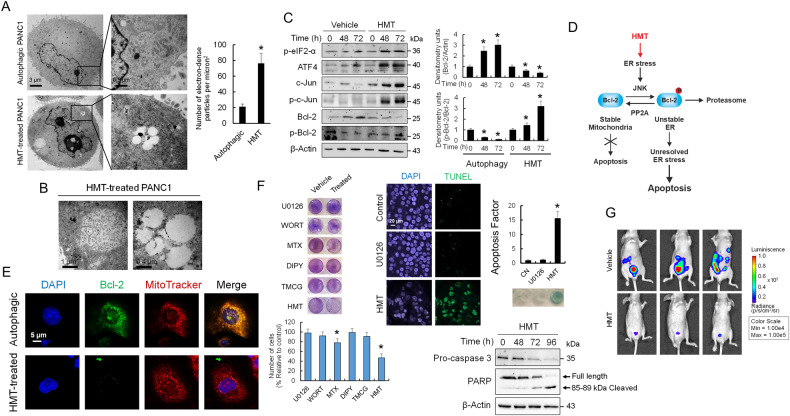
HMT induces ER stress-dependent apoptosis in PANC1 cells. **A** TEM image showing ribosomes in autophagic (vehicle treatment) and HMT-treated (72 h) PANC1 cells. The boxed area contains representative ribosomes at ×60,000 magnification. Ribosomes were quantified using ImageJ as indicated in the “Methods” section. *P < 0.05 compared with untreated controls. **B** PANC1 cells were treated with HMT for 72 h and then analyzed by TEM. They exhibited vacuolation of the cell cytoplasm due to swelling of the ER cisternae. **C** Western blots showing the expression of the indicated proteins in PANC1 cells after 72 h of vehicle or HMT treatment. The histograms show the relative amounts of Bcl-2 and p-Bcl-2 versus β-actin and Bcl-2, respectively, under different treatments. *P < 0.05 compared with untreated controls. **D** Schematic representation of HMT inducing ER stress-dependent apoptosis. **E** Confocal microscopy showing Bcl-2 and mitochondrial localization in PANC1 cells. **F** HMT significantly decreased cell viability in PANC1 cells compared with individual treatments [TMCG (10 µM) or DIPY (5 µM)] and those including U0126 (10 µM), wortmannin (WORT; 0.2 µM), or methotrexate (MTX, 10 µM). Cells were subjected to the indicated treatment for 72 h. Following HMT treatment, a reduction in cell viability was accompanied by an increase in apoptosis (as determined by TUNEL) and the cleavage of PARP and caspase 3 (as indicated by a time-dependent reduction in full-length caspase 3). Histograms represent the apoptotic factor (assuming an apoptotic factor of 1 for PANC1 untreated cells) evaluated using a DNA fragmentation assay. *P < 0.05 compared with untreated controls. **G** Luciferase imaging of vehicle (control) and HMT-treated mice at 21 days post-tumor cell injection (*N* = 3). Firefly luciferin (120 mg/kg) was injected intraperitoneally.

Impaired autophagy contributes to ER stress by inhibiting the degradation of misfolded proteins, which undergo ubiquitination and are bound to p62/SQSTM1 (Fig. 1C) [32]. Interestingly, and in agreement with a hyperactive mTORC1 pathway in HMT-treated cells resulting in active S6K1 (Fig. 3A), there was a significant increase in ribosomes associated with the ER in HMT-treated cells when compared with autophagic cells (Fig. 6A). The HMT-treated cells also showed characteristics of early stage-ER stress, including a distended and dilated ER and vacuolation of the cell cytoplasm due to swelling of the ER cisternae (Fig. 6B). Taken together, these data indicated that HMT gives controversial instructions to pancreatic cancer cells, while force them to continuous protein synthesis, but suppressing autophagy mechanisms to removal misfolded proteins (Fig. 2A).

Consequently, we observed increased expression of ER stress markers, such as eIF2α phosphorylation and ATF4, in HMT-treated PANC1 cells compared with vehicle-treated controls (Fig. 6C). ER stress-induced autophagy switches to apoptosis under accumulative damage stimuli. Here, we observed that HMT activated c-Jun N-terminal kinase (JNK) signaling (Fig. 6C), an indicator of severe and unresolved ER stress [33]. The activation of JNK by HMT led to the phosphorylation and stabilization of c-Jun while eliminating the antiapoptotic effect of Bcl-2 (Fig. 6C). Interestingly, phosphorylation of Bcl-2 by JNK at the ER is required for its proteasome-dependent degradation, while PP2A-mediated dephosphorylation of Bcl-2 protects it from degradation and induces its mitochondrial translocation, favoring cell resistance to several classes of death stimuli (including ER stress) (Fig. 6D) [33, 34]. Although PANC1 cells had increased Bcl-2 levels during spontaneous autophagy, probably to protect against apoptosis, the HMT-treated cells showed reduced levels of Bcl-2, and it was mainly in its phosphorylated state. Confocal microscopy also revealed different localizations of Bcl-2 in autophagic and HMT-treated cells (Fig. 6E). Since HMT-induced JNK signaling while inhibiting PP2A activity, treatment of pancreatic cancer cells with this combination had a knock-on proapoptotic effect that resulted in caspase-mediated cell death (Fig. 6F). Next, we assayed HMT activity in a mouse model of peritoneal carcinomatosis. For this, PANC1-luc cells were injected intraperitoneally into female nude mice (Hsd: athymic nude-Foxn1nu), and the evolution of tumors was evaluated using an IVIS Lumina-2 system. Compared with vehicle treatment, HMT significantly reduced tumor growth (Fig. 6G).

## Discussion

Recent studies indicated that combined inhibition of EGFR and CRAF in KRAS-driven models of PDAC prevented tumor development [35]; therefore, there is a growing interest in targeting CRAF as a strategy to treat KRAS mutant tumors [36]. Although drugs that directly target CRAF have not yet been successfully developed, other strategies to prevent CRAF activation have been proposed [30]. Since CRAF activation (by dephosphorylation of S259) depends on a functional SHOC2 complex [28], the indirect modulation of CRAF by interfering with the components of this complex (SHOC2, MRAS, and PP1) could be of therapeutic interest. In fact, the identification of both SHOC2-dependent and -independent mechanisms of ERK activation [28] could help to elucidate the importance of blocking the MAPK pathway at the level of CRAF. Since aberrant signaling in KRAS-mutant cancer cells is entirely dependent on the SHOC2-dependent pathway, this suggests that, in contrast to targeting MEK or ERK (which inhibit global ERK signaling), blocking SHOC2 function would interfere only with oncogene-driven ERK activation and would not affect other aspects of normal ERK regulation mediated through SHOC2-independent mechanisms, which would bypass KRAS membrane activation [28]. Since a residual p-ERK2 pathway is necessary to induce ATF4-dependent cell death [37], and disruption of SHOC2 would only affect oncogenic KRAS signaling, the blockade of CRAF activation by disrupting the SHOC2 complex might be a therapeutic route to induce apoptosis in tumor cells but with less toxicity for the patient.

In this context, our results show that simultaneous blocking of the MAPK pathway and autophagy under hypomethylating conditions was more effective in inducing apoptosis in pancreatic cancer cells than the application of specific drugs, such as U0126 and wortmannin, which are designed to inhibit the MAPK and PI3K pathways, respectively (Fig. 6F). Our study points to MRAS as an attractive target in PDAC. Unlike other components of the MAPK pathway, such as KRAS, BRAF, and NRAS, MRAS has not been found to be mutated in human cancers [30], and perhaps this circumstance has excluded it from being an attractive anticancer target. However, its relevant function during CRAF activation, together with the fact that it is not constitutively active and is not permanently located in the cell membrane, could generate new therapeutic opportunities. The observation that HMT compromises BRAF stability could also be of interest to explain the effect of these hypomethylating therapies on BRAF-mutated melanomas [38, 39].

**Fig. 7:**
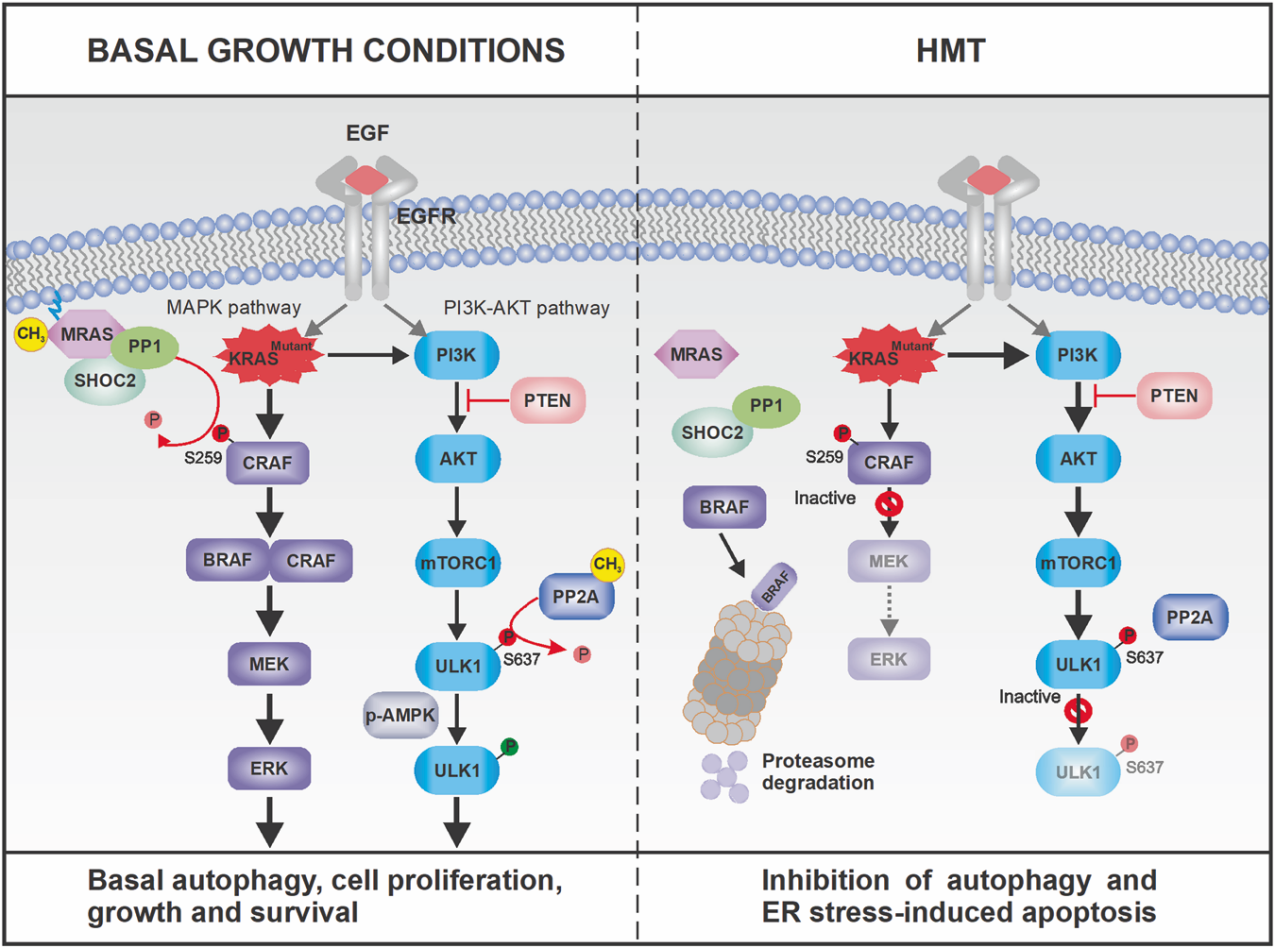
Proposed mechanism(s) by which impaired protein methylation inhibits autophagy in KRAS-mutated PDAC tumors. PDAC cell growth depends on high basal autophagy [18]. In addition to mTORC1 inactivation, starvation also causes an increase in PP2A activity toward ULK1, a mTORC1 substrate whose dephosphorylation at S637 is required for autophagy induction. Strong PP2A activity toward MiT/TFE proteins and ULK1 can explain the contradictory coexistence of strong basal autophagy activity and intact mTORC1 function in KRAS-mutated PDAC tumors. Under such conditions, activation of MRAS by protein methylation maintains and operates the RAS–MEK–ERK pathway to ensure cell proliferation, growth and survival. However, mechanisms for maintaining autophagy and cell survival are disrupted under hypomethylating conditions (HMT). Impaired protein methylation induced a KRAS signaling imbalance that resulted in attenuation of the MAPK pathway but hyperactivation of the PI3K/AKT pathway. The high levels of AKT activation (by phosphorylation at S473) in the presence of HMT failed to inhibit mTORC1 even in the presence of its specific inhibitor rapamycin. Therefore, by activating mTORC1 while inhibiting the methylation/activation of PP2A, HMT induced strong autophagy inhibition, which has been found to protect KRAS-mutated PDAC tumors from drugs designed to specifically target the MAPK pathway [6–8]. Residual ERK2 activation maintained by SHOC2-independent mechanisms together with the disruption of PP2A-dependent control of anti-apoptotic Bcl-2 rendered the pancreatic cancer cells unable to defend themselves against cumulative damage resulting from HMT-induced ER stress.

Importantly, an HMT-induced decrease in PP2A activity may also lead to the failure of nuclear translocation of MiT/TFE factors and a decrease in ULK1 activity for autophagy activation. On the one hand, hypomethylation conditions restore mTORC1-mediated suppression of MiT/TFE factors [21], which disables PDAC cells to maintain robust activation of anabolic pathways while simultaneously benefiting from autophagy/lysosomal pathway-mediated catabolic process [21]. On the other hand, the indirect effect of HMT on the kinase activity of ULK1 suggests that this treatment inhibits autophagy by disrupting the maturation of autophagosomes [40]. The relationship between KRAS-MAPK signals and autophagy is described in Fig. 7 and we noticed that this perfectly orchestrated mechanism for maintaining basal autophagy and cell survival in PDAC was completely disrupted under hypomethylating conditions. Therefore, the use of HMT alone, in the presence of MEK/ERK inhibitors, or strategies to disrupt protein homeostasis by jointly blocking protein degradation in the lysosomes and the ubiquitin-proteasome system are promising strategies that could be explored for the treatment of PDAC tumors. The results of these further studies will allow us to draw conclusions about whether protein methylation constitutes the Achilles’ heel of pancreatic tumor cells and whether therapies directed at the epigenetics of these cells could be transferred to the clinic.

## Methods

### Reagents and antibodies

TMCG was synthesized from catechin via reaction with 3,4,5-trimethoxybenzoyl chloride [41]. DIPY, rapamycin, AdOx, MTX, U0126, wortmannin, IAKT VIII trifluoroacetate salt hydrate, MG132, and EGF were obtained from Merck (Madrid, Spain). Antibodies against the following proteins were used: β-actin (Merck; monoclonal clone AC-15), AKT (Abcam, Cambridge, UK; monoclonal clone EPR17737), ATF4 (Cell Signaling Technology, Danvers, MA, USA; polyclonal H-277), Beclin-1 (Thermo-Fisher; monoclonal), Bcl-2 (Merck; monoclonal), BRAF (Abcam; monoclonal clone EP152Y), caspase 3 (Santa Cruz Biotechnology, Dallas, TX, USA; monoclonal), c-Jun (Thermo-Fisher; recombinant polyclonal clone 2HCLC), CRAF (Thermo-Fisher; polyclonal), EGFR (Abcam; monoclonal clone EP38Y), ERK1/2 (Santa Cruz Biotechnology; monoclonal), GADPH (Abcam; monoclonal), Lamp-1 (Abcam; polyclonal), LC3B (Abcam, polyclonal), MEK1/2 (Thermo-Fisher; polyclonal), metyl-PP2Ac (Merck; monoclonal clone 2A10), MITF (Merck; monoclonal clone C5), MRAS (Thermo-Fisher; monoclonal clone OTI3C4), MRAS (Abcam; monoclonal clone EPR12457), p62/SQSTM1 (Thermo-Fisher; polyclonal), p-AKT-S473 (Merck; monoclonal clone 6F5), p-AKT-T308 (Santa Cruz Biotechnology; monoclonal), PARP (Abcam; monoclonal clone E102), p-Bcl-2-S70 (Merck; polyclonal), p-c-Jun (Thermo-Fisher; polyclonal), p-CRAF-S259 (Thermo-Fisher; polyclonal), p-eIF2α (Cell Signaling Technology; monoclonal), p-ERK1/2 (Merck; monoclonal), PGC-1α (Santa Cruz Biotechnology; monoclonal), p-MEK1/2-S217-S221 (Thermo-Fisher; monoclonal clone C.158.9), PP2Ac (Merck; monoclonal clone 1D6), p-S6K-T389 (Merck; polyclonal B2HCLC), p-ULK1-S638 (Abcam; monoclonal clone EPR6155), S6K (Merck; monoclonal clone 5G9), SHOC2 (Merck; polyclonal), TATA binding protein (TBP: Abcam; monoclonal), TFE3 (Merck; polyclonal), TFEB (Merck; polyclonal), and ULK1 (Merck; polyclonal).

### Cell cultures and treatments

PANC1 cells constitute a cellular model for PDAC with mutated *KRAS* (heterozygous p. Gly12Asp; c.35G>A) and *TP53* (homozygous p. Arg273His; c.818G>A). To extend our analysis to other PDAC models, we studied the effect of HMT on Hs766T cells, which are homozygous for *KRAS* (p. Gln61His; c.183A>C) but have no *TP53* mutation. PANC1 and Hs766T cell lines were obtained from ATCC and tested for mycoplasma and authenticated using genotype profiling according to the ATCC guidelines. The cells were cultured in Dulbecco’s modified Eagle’s medium supplemented with 10% fetal bovine serum, 1 mM pyruvate, and 2 mM glutamine and incubated at 37 °C, 7.5% CO_2_ and 95% humidity. Penicillin and streptomycin were added to the culture medium at final concentrations of 50–100 IU/ml and 50–100 μg/ml, respectively. Basal autophagy, defined here as macroautophagic activity during cell growth in a normal medium containing amino acids and serum, appeared to be highly active in the pancreatic cancer cells used in this study. To study basal autophagy, approximately 60–70% of confluent cells were seeded 24 h before drug treatments. Resting cells (24 h after seeding) were treated (zero time) with vehicle (DMSO) or HMT (consisting of a combination of 10 μM TMCG and 5 μM DIPY in DMSO) to evaluate basal autophagy and HMT-related effects, respectively, during the indicated times. When indicated, autophagic cells (72 h under vehicle treatment) and HMT-treated cells (72 h under HMT treatment) were stimulated with 30 ng/ml EGF for 5 min.

### Cell proliferation measurement

Cell proliferation was evaluated using colorimetric assays to analyze mitochondrial function (MTT; Merck). This assay is based on the ability of the metabolically active cell enzyme succinate dehydrogenase to convert MTT to water-insoluble blue-colored formazan. The amount of formazan is directly proportional to the number of cells. For the assay, culture medium was removed from 96-well plates, and 0.2 ml of fresh medium was added to each well. Next, 50 μl of MTT solution (5 mg/ml dissolved in culture medium) was added and incubated in the dark for 2 h. The medium was then removed, and 100 μl of DMSO was added to solubilize the formed formazan. The absorbance was measured at 570 nm in a FLUOstar Omega plate reader spectrophotometer (BMG Labtech, Ortenberg, Germany) using 690 nm as the reference wavelength. In other cases, the % surviving attached cells (% crystal violet OD) was used to compare vehicle control versus treated cells. For this, the cells were washed twice with 1x PBS and stained with 0.5% crystal violet (in 20% methanol) for 20 min. The cells were then washed with water 4 times, and the plates were air-dried overnight. The next day, 200 μl of DMSO was added to each well and incubated for 20 min at room temperature. The optical density was measured at 590 nm. Experiments were performed in triplicate at least three times.

### Metabolite measurements

Intracellular metabolites were isolated on ice by sonication of 10 × 10^6^ cells in 1 ml of ice-cold PBS using a 30 kHz sonicator with the probe at 30% amplitude for three 20-s cycles with 1 min breaks in between. The resultant cell-free supernatants were snap-frozen and stored at −80 °C. Quantification of intracellular SAM and SAH concentrations was then conducted using the SAM and SAH ELISA Combo Kit from Cell Biolabs Inc. (STA-671-C; San Diego, CA, USA) following the manufacturer’s protocol.

### Microscopy

Laser-scanning CM of fixed cells was performed using a Leica TCS 4D confocal microscope (Wetzlar, Germany). For indirect immunofluorescence studies, cells were grown on 100-mm^2^ coverslips and fixed with acetone. Coverslips were incubated in 5% BSA for 20 min and probed with primary antibodies (diluted 1:200 in PBS containing 5% BSA) for 2 h at room temperature. The cells were then washed three times in PBS and incubated for 1 h at room temperature with Alexa Fluor dyes (Thermo-Fisher). The coverslips were permanently mounted to the slides using a fluorescence mounting medium (ProLong-Gold, Thermo-Fisher). Nuclei and lysosomes were stained with DAPI and LysoTracker Red DND-99, respectively. Cathepsins were labeled with an iABP probe (iABP Pan Cathepsin Probe, Vergent Bioscience, Minneapolis, MN, USA) specifically designed to label cathepsins B/L/S/X. For TEM, cells were centrifuged for 10 min at 1500 rpm and fixed with 3% glutaraldehyde in 0.1 M cacodylate buffer (1 h at 4 °C). Next, the fixative was removed by centrifugation, and the samples were left overnight at 4 °C in 0.1 M cacodylate buffer containing 8% sucrose. After centrifuging again, the buffer was removed, and the samples were placed in 1% osmium tetroxide (2.5 h at 4 °C). After removing the osmium tetroxide by centrifugation, the samples were resuspended in 0.1 M cacodylate buffer containing 8% sucrose. The samples were then dehydrated with ethanol in increasing concentrations (30–100%), washed with propylene oxide, and embedded in epoxy resin (EPON). Finally, the samples were cut into ultrathin sections with an ultramicrotome and stained with lead citrate and uranyl acetate. The preparations were visualized on a JEOL 1011 transmission electron microscope (JEOL, Inc., Peabody, MA, USA), and photographs were taken with a Gatan Orius SC200 high-contrast coupled digital camera (Gatan, Inc., Pleasanton, CA, USA). For the quantification of ribosomes and lysosomes, electronic images were processed with a modification of a previously described method [42]. Images were contrast-enhanced in Adobe Photoshop (Adobe Systems, Inc., San Jose, CA, USA) for clarity and subsequently clean masks were analyzed with the “Analyze particles” routine of ImageJ 1.37c25 (National Institutes of Health, Bethesda, MD) to obtain the estimated number of ribosomes and lysosomes.

### Stealth RNA transfections

Specific stealth siRNAs for SHOC2 (HSS111928) and PP2A-Cα (HSS108358) were obtained from Thermo-Fisher and transfected into PANC1 cells using Lipofectamine 2000 (Thermo-Fisher). Stealth RNA-negative control duplexes (Thermo Fisher) were used as control oligonucleotides, and the ability of the stealth RNA oligonucleotides to knockdown the expression of selected genes was analyzed using Western blot analysis at 72 h after siRNA transfection.

### Western blots

Whole-cell lysates were collected in SDS‒PAGE sample loading buffer. After sonication, the samples were boiled (10 min), and proteins were separated by SDS‒PAGE and then transferred to nitrocellulose membranes and analyzed using immunoblotting (WesternBright Quantum, Advansta, San Jose, CA, USA).

### Immunoprecipitation

For immunoprecipitation assays, the cells (~5 × 10^6^) were lysed in 500 μl of lysis buffer (50 mM Tris, pH 8.0, 300 mM NaCl, 0.4% NP40, 10 mM MgCl_2_) supplemented with protease and phosphatase inhibitor cocktails (Merck). The cell extracts were cleared by centrifugation (20,000×*g* for 15 min) and then diluted with 500 μl of dilution buffer (50 mM Tris, pH 8.0, 0.4% NP40, 2.5 mM CaCl_2_) supplemented with protease and phosphatase inhibitor cocktails and DNase I (Merck). The extracts were precleared in 30-min incubations with 20 μl of Pure Proteome Protein G Magnetic Beads at 4 °C while being rotated. The antibodies (as indicated in the figure legends) were incubated with 50 μl of Pure Proteome Protein G Magnetic Beads for 1 h at RT. After extensive washing, Protein G Magnetic Beads containing antibodies were washed, and the precleared extracts were added and incubated overnight at 4 °C with rotation. After extensive washing, the bound proteins were analyzed by Western blot. The unbound extracts were used as the positive inputs to determine protein loading.

### Cell fractionation

The plasma membrane fraction was isolated using a plasma membrane protein extraction kit (Abcam; ab65400) following the manufacturer’s instructions. In brief, cells were lysed in homogenization buffer containing a protease inhibitor cocktail. The homogenates were centrifuged at 700×*g* for 10 min at 4 °C. Pellets containing plasma membrane and organelle membranes were isolated from the cytosol fraction by high-speed centrifugation of the supernatants at 10,000×*g* for 30 min at 4 °C. To isolate the plasma membrane fraction further, pellets were resuspended in the upper phase buffer and extracted in the lower phase buffer. This was followed by centrifugation to pellet the plasma membrane fraction. The plasma membrane pellets were solubilized in 0.5% Triton X-100 in PBS for Western blotting. Cytosolic extracts were obtained using NE-PER Nuclear and Cytoplasmic Extraction Reagents (Thermo-Fisher).

### PP2A assay

The PP2A assays were performed as previously described [43]. After two washes with 0.9% NaCl, total cellular proteins were extracted in lysis buffer containing 50 mM Tris–HCl (pH 7.5), 250 mM NaCl, 3 mM EDTA, 3 mM EGTA, 1% Triton X-100, and 0.5% NP-40 without phosphatase inhibitors. Specific PP2A activity was measured using the PP2A Immunoprecipitation Phosphatase Assay Kit (Merck). All procedures were performed according to the manufacturer’s protocol, and changes in absorbance were measured at 650 nm in a Spectra-MAX 250 plate reader (Molecular Devices, Sunnyvale, CA, USA).

### Quantification of autophagosomes

Autophagosomes were quantified using an LC3B Antibody Kit for Autophagy (Thermo-Fisher). PANC1 or Hs766T cells (5 × 10^5^) were treated as indicated, fixed with methanol, and stained with a green detection reagent. Images were captured with a fluorescence microscope. The total number of green dots per field was counted in five different images per sample from the treated/untreated groups. The number of autophagosomes/100 cells was calculated. In parallel, cells were treated with vehicle (with and without CQ) and processed similarly, which served as positive controls.

### Apoptosis assays

Analysis of apoptotic cells was performed using the terminal deoxynucleotidyl transferase-mediated dUTP nick-end labeling (TUNEL) staining kit following the manufacturer’s instruction (Roche Diagnostics). Images of cells were taken using a fluorescence microscope. Apoptosis was quantified by ELISA (Cell Death Detection ELISA PLUS, Roche Diagnostics, Barcelona, Spain) to detect mono- and oligonucleosomes in the cytoplasmic fractions of cell lysates using biotinylated antihistone and peroxidase-coupled anti-DNA antibodies. The amount of nucleosomes was photometrically quantified at 405 nm by determining the peroxidase activity that was retained in the immunocomplexes. Apoptosis was defined as the specific enrichment of mono- and oligonucleosomes in the cytoplasm and calculated by dividing the absorbance of the treated samples by the absorbance of the untreated samples after correcting for the number of cells.

### RAS activation assays

KRAS activity was assayed using a KRAS Activation Assay Kit (Cell Biolabs, Inc.) according to the manufacturer’s protocol. Briefly, PANC1 cells (2 × 10^7^ per sample) were lysed in 1 ml of lysis buffer (25 mM HEPES, pH 7.5, 150 mM NaCl, 1% NP‐40, 10 mM MgCl_2_, 1 mM EDTA, 2% glycerol), and the lysates were incubated with CRAF RBD agarose beads for 3 h at 4 °C. The beads were collected by centrifugation and washed thrice with assay buffer. KRAS was eluted from the beads with Laemmli sample buffer. The samples were subjected to SDS‐PAGE, and the pull‐down of active KRAS was assessed by Western blotting with an anti‐KRAS antibody. In the RAS activation assay kit for ELISA (Cat. #17-497; EMD Millipore), 200 µg of total protein was used at 1 µg/µL, and the RAS-GTP pull-down was measured using a microplate reader (Fluostar-Omega, BMG Labtech, Ortenberg, Germany) in luminescent mode.

### Mice

Female athymic nude-Foxn1nu mice, 4–5 weeks of age, were obtained from Envigo (Barcelona, Spain). The mice were housed under aseptic conditions (positive air pressure in a designated mouse room with microisolator tops) in the specific pathogen-free animal facility at the University of Murcia, and all mouse handling procedures were carried out under a laminar flow hood. Primary tumors were established intraperitoneally with 5 × 10^5^ PANC1 cells expressing luciferase. HMT was administered intraperitoneally on Day 5 and consisted of a mixture of TMCG (30 mg/kg/day) and DIPY (10 mg/kg/day). Control mice received the same volume of vehicle (DMSO). Peritoneal carcinomatosis was analyzed using the IVIS Imaging System (Caliper Life Sciences, Hopkinton, MA, USA). We made every attempt to reach conclusions using as small sample size as possible. We usually exclude samples if we observe any abnormality in terms of size, weight, or apparent disease symptoms in the animal before performing experiments. However, we did not exclude any animals in the present study, as we did not observe any abnormalities. Neither randomization nor blinding was performed in this study.

### Quantification and statistical analysis

The Western blot analyses and the microscopy data analyses were performed as previously described [10]. The analyses were repeated at least three times, and they showed similar results. The results from one experiment are shown. To quantify the results, the Western blots were scanned using a Bio-Rad ChemiDoc scanning densitometer (Bio-Rad Laboratories), and relative band intensities were analyzed using a Gel-Pro Analyzer (Media Cybernetics Inc., Rockville, MD, USA). For other experiments, the mean ± SD of three measurements performed in triplicate was calculated. Numerical data were analyzed to determine statistical significance using Mann–Whitney tests for comparisons of means in SPPS statistical software for Microsoft Windows, release 6.0 (Professional Statistic, Chicago, IL, USA). Individual comparisons were made using Student’s two-tailed, unpaired *t*-tests. The criterion for significance was *P* < 0.05 for all comparisons.

### Supplementary information

Reproducibility Checklist

Full and uncropped western blots

## Data Availability

All datasets generated and analysed during this study are included in the published article. Additional data are available from the corresponding author on reasonable request.
