# Supplementary material for: Targeting protein methylation in pancreatic cancer cells results in KRAS signaling imbalance and inhibition of autophagy
Source: Cell Death Dis. 2023 Nov 23;14(11):761. doi: 10.1038/s41419-023-06288-9 (PMC10667277; doi:10.1038/s41419-023-06288-9)

Full and uncropped western blots

Targeting protein methylation in pancreatic cancer cells results in KRAS signaling imbalance and inhibition of autophagy

María F. Montenegro[1,4]✉, Román Martí-Díaz[1,4], Ana Navarro[2], Jorge Tolivia[2], Luis Sánchez-del-Campo[1], Juan Cabezas-Herrera[3], and José Neptuno Rodríguez-López[1]✉

Fig. 1A

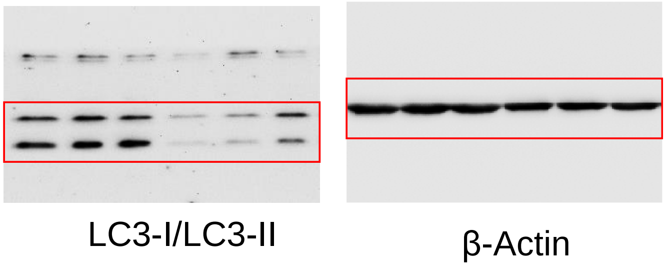

Fig. 1C

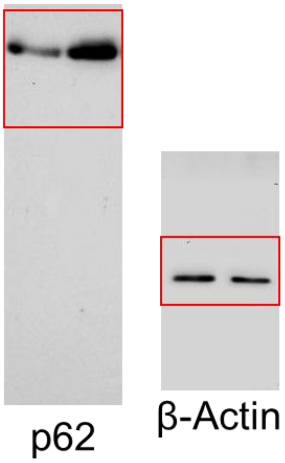

Fig. 1E

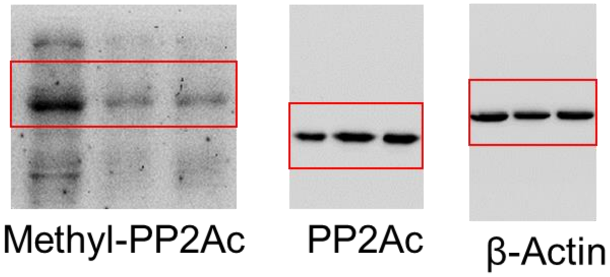

Fig. 2B

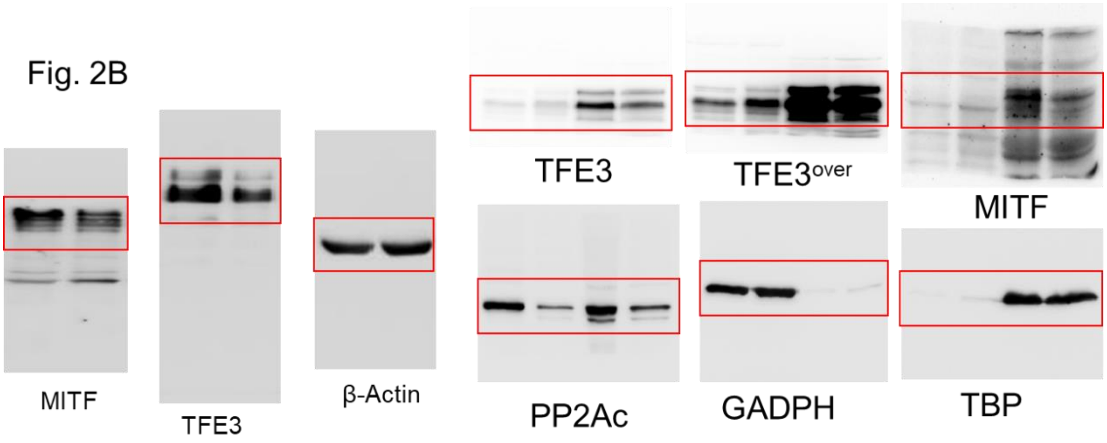

Fig. 2D

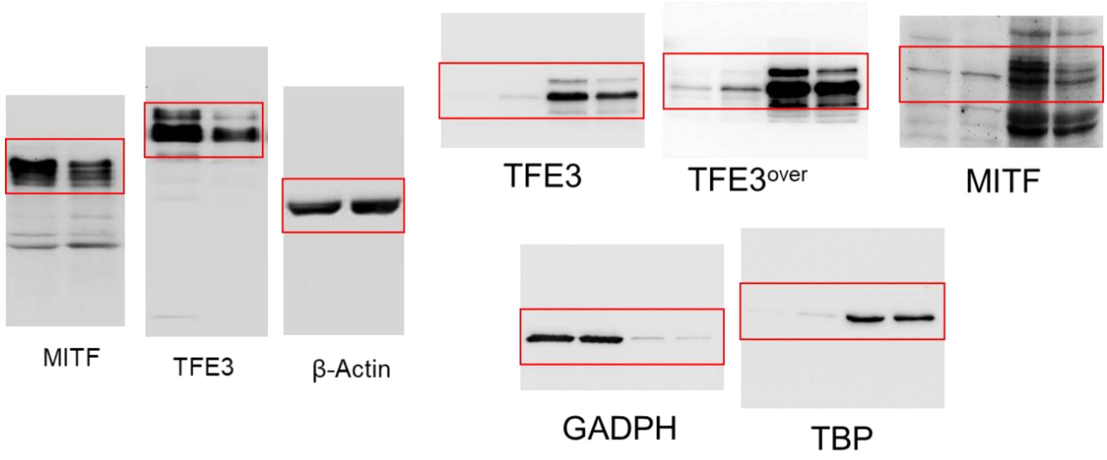

Fig. 2E

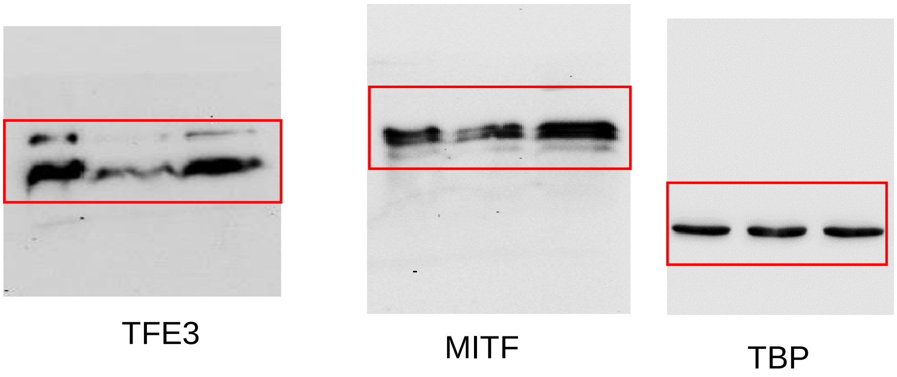

Fig. 2F

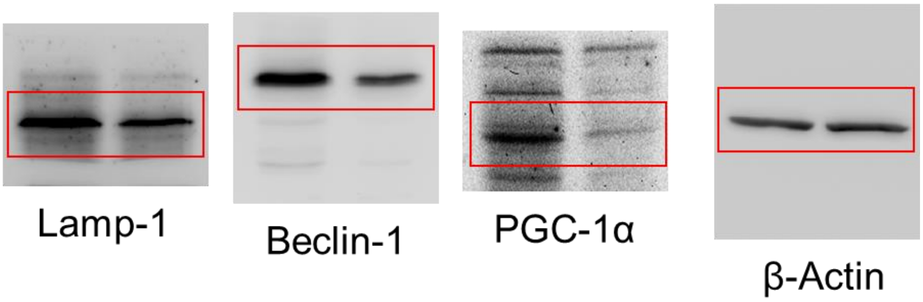

Fig. 3A

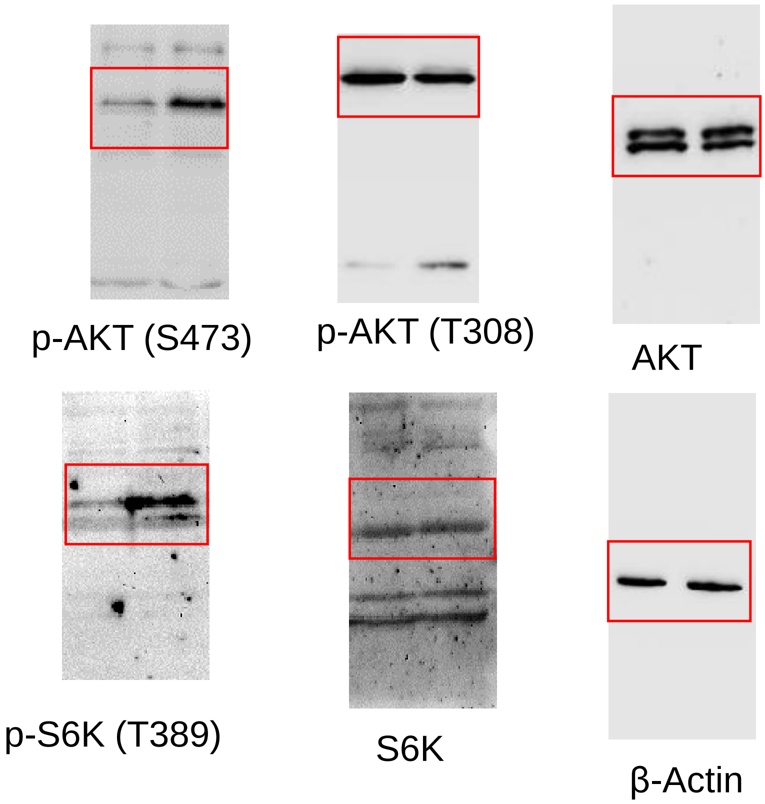

Fig. 3B

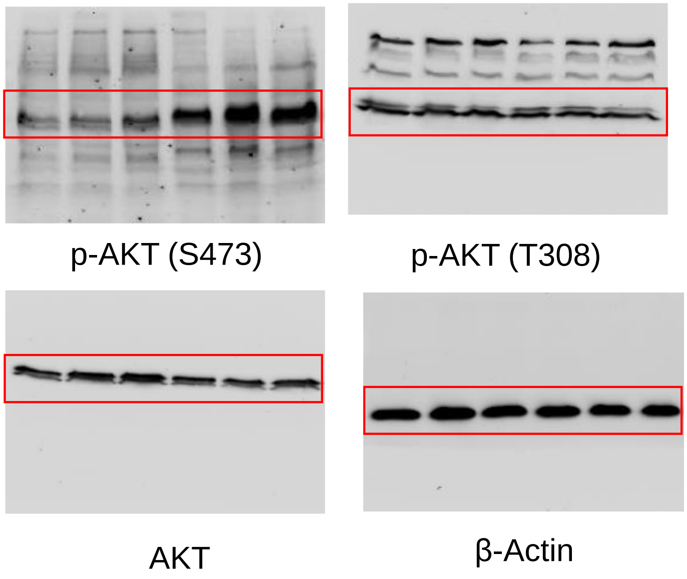
p-AKT (S473)
p-AKT (T308)
AKT
β-Actin

Fig. 3C

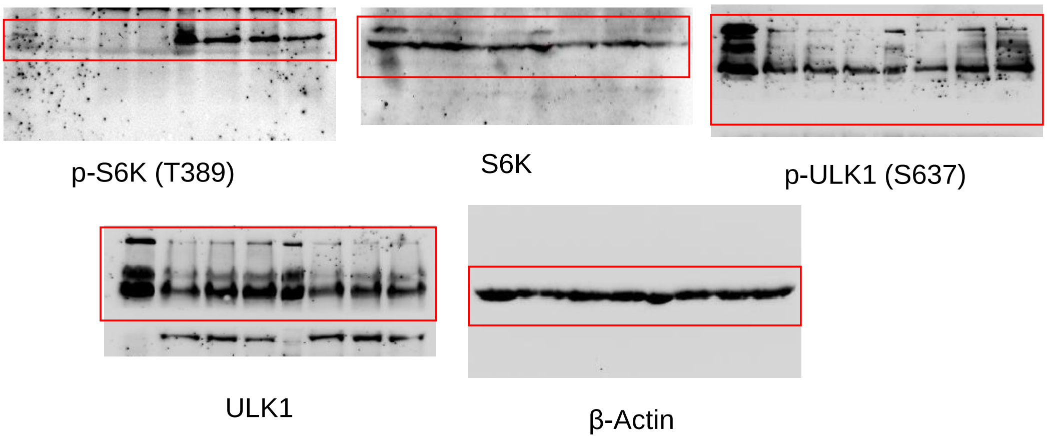
p-S6K (T389)
S6K
p-ULK1 (S637)
ULK1
β-Actin

Fig. 3D

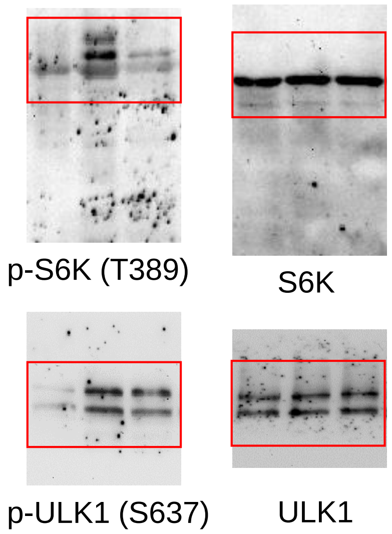

Fig. 4A

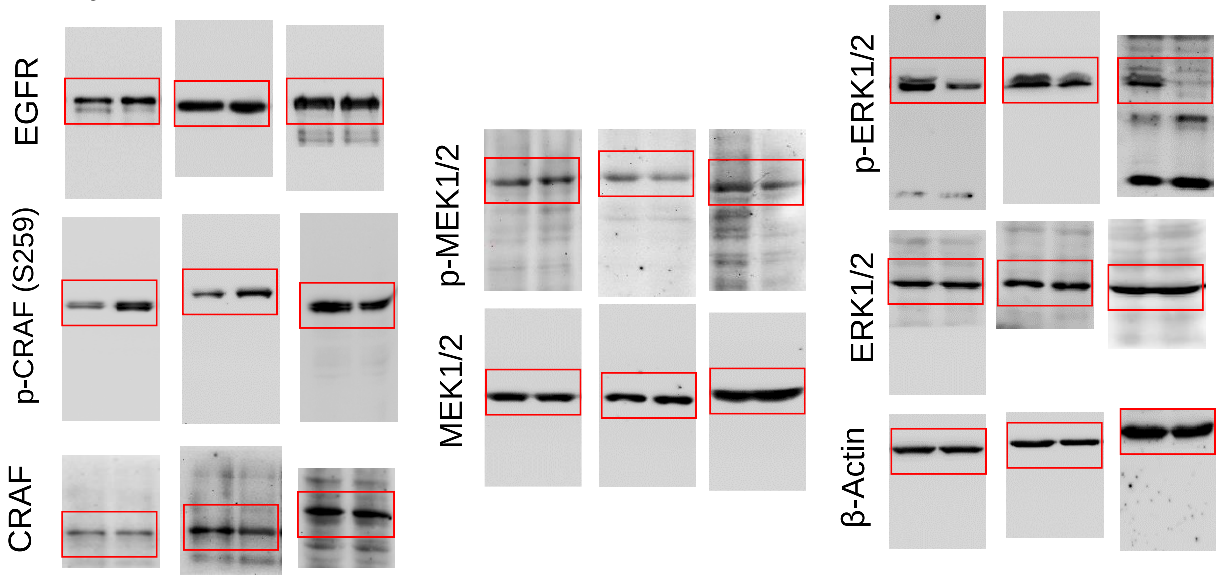

Fig. 4B

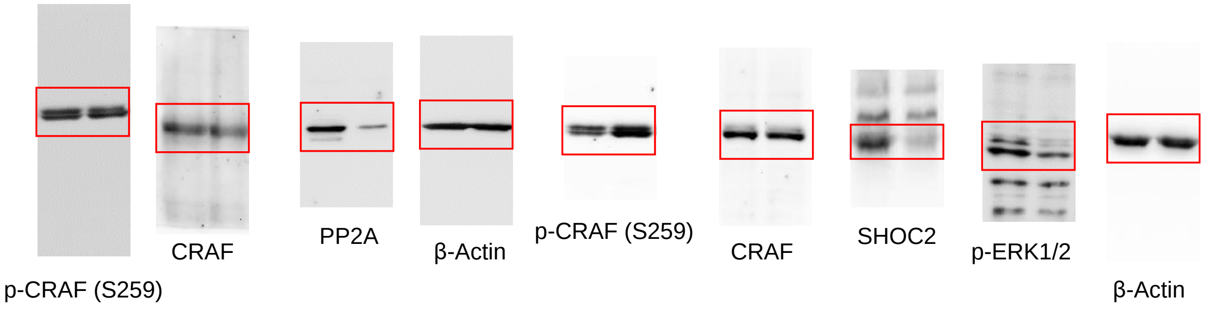

Fig. 4C

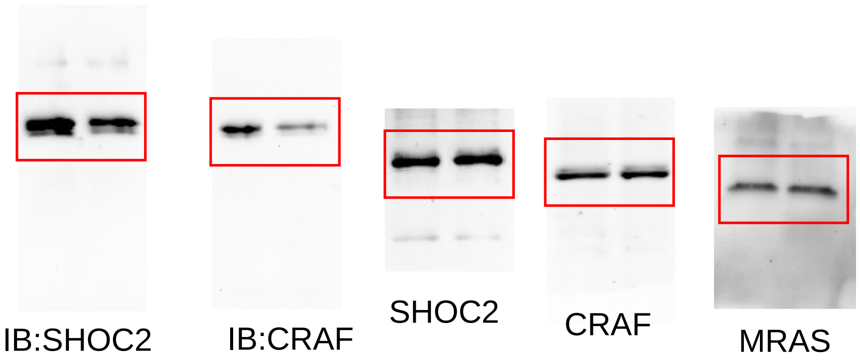

Fig. 4E

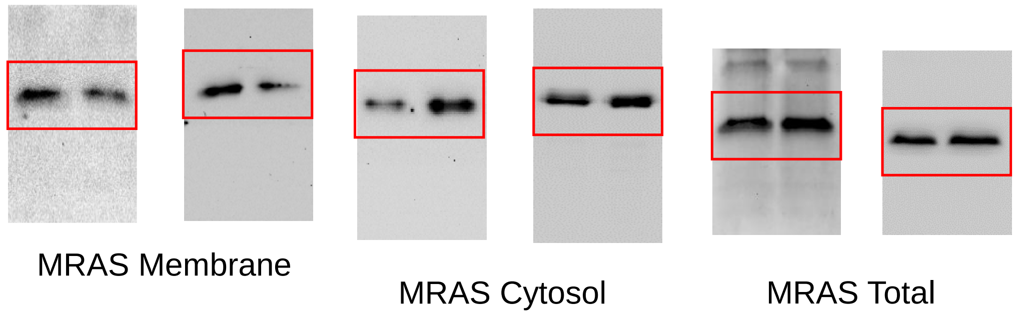

Fig. 4F

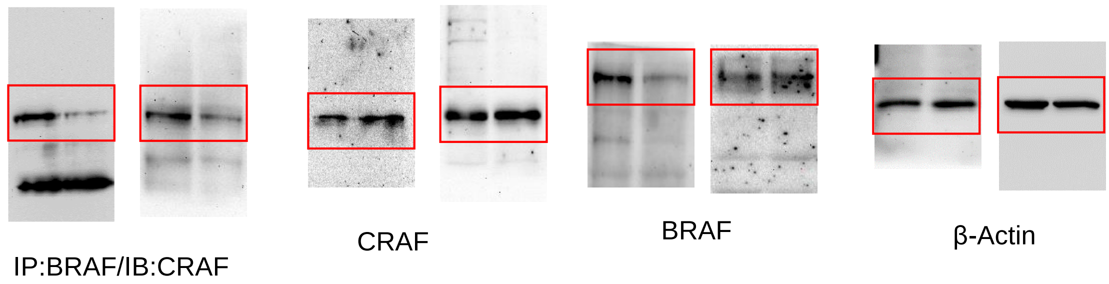

Fig. 4G

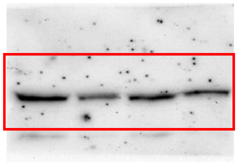

BRAF

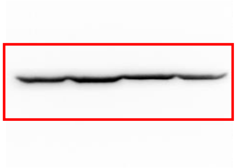

β-Actin

Fig. 5B

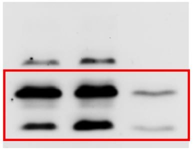

LC3-I/LC3-II

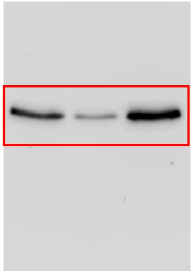

p62

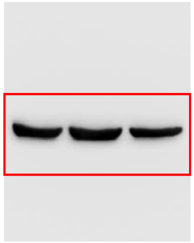

β-Actin

Fig. 5C

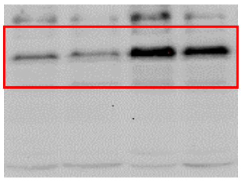

p-AKT (S473)

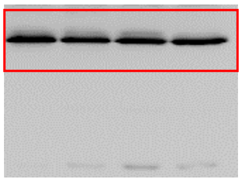

p-AKT (T308)

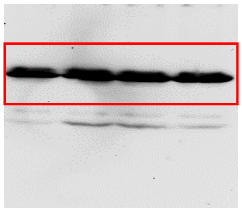

AKT

Fig. 5D

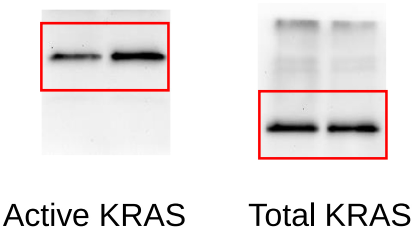

Fig. 6C

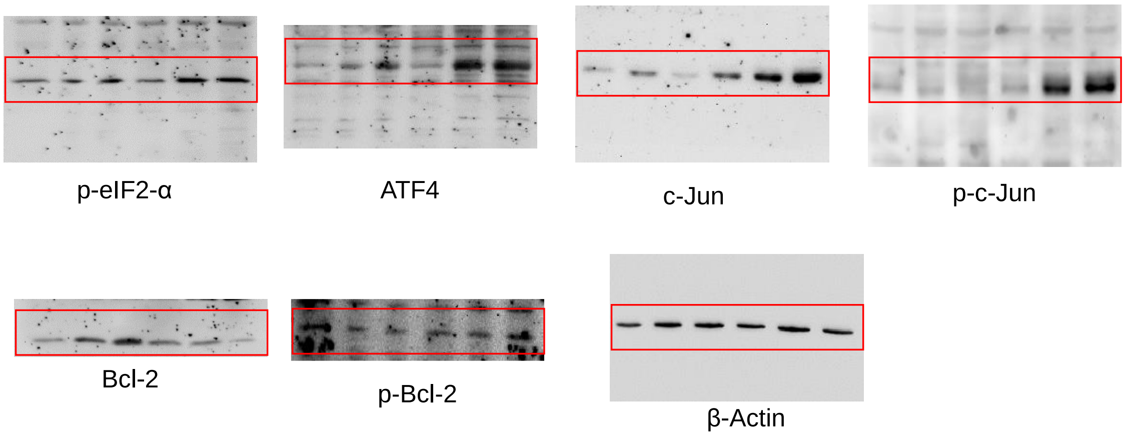

Fig. 6F

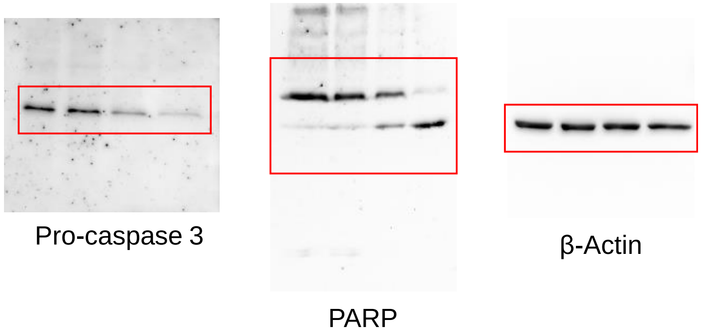

Supplement: Supplementary file 2 — Full and uncropped western blots [file 41419_2023_6288_MOESM2_ESM.pdf]
